# Evolutionary constraints and expression analysis of gene duplications in *Rhodobacter sphaeroides* 2.4.1

**DOI:** 10.1186/1756-0500-5-192

**Published:** 2012-04-25

**Authors:** Anne E Peters, Anish Bavishi, Hyuk Cho, Madhusudan Choudhary

**Affiliations:** 1 Department of Biological Sciences, Sam Houston State University, Huntsville, TX 77341, USA; 2 Department of Computer Science, Sam Houston State University, Huntsville, TX 77341, USA

**Keywords:** Gene duplication, In-paralog, Out-paralog, Evolutionary constraint, Microarray, Gene expression, *Rhodobacter sphaeroides*

## Abstract

**Background:**

Gene duplication is a major force that contributes to the evolution of new metabolic functions in all organisms. *Rhodobacter sphaeroides* 2.4.1 is a bacterium that displays a wide degree of metabolic versatility and genome complexity and therefore is a fitting model for the study of gene duplications in bacteria. A comprehensive analysis of 234 duplicate gene-pairs in *R. sphaeroides* was performed using structural constraint and expression analysis.

**Results:**

The results revealed that most gene-pairs in in-paralogs are maintained under negative selection (*ω* ≤ 0.3), but the strength of selection differed among in-paralog gene-pairs. Although in-paralogs located on different replicons are maintained under purifying selection, the duplicated genes distributed between the primary chromosome (CI) and the second chromosome (CII) are relatively less selectively constrained than the gene-pairs located within each chromosome. The mRNA expression patterns of duplicate gene-pairs were examined through microarray analysis of this organism grown under seven different growth conditions. Results revealed that ~62% of paralogs have similar expression patterns (cosine ≥ 0.90) over all of these growth conditions, while only ~7% of paralogs are very different in their expression patterns (cosine < 0.50).

**Conclusions:**

The overall findings of the study suggest that only a small proportion of paralogs contribute to the metabolic diversity and the evolution of novel metabolic functions in *R. sphaeroides*. In addition, the lack of relationships between structural constraints and gene-pair expression suggests that patterns of gene-pair expression are likely associated with conservation or divergence of gene-pair promoter regions and other coregulation mechanisms.

## Background

*Rhodobacter sphaeroides* is a well-studied photosynthetic eubacterium that belongs to the α-3 subgroup of the *Proteobacteria*[1,2]. *R. sphaeroides* 2.4.1 is a model strain for this organism and is noteworthy since its genome consists of two chromosomes, chromosome I (CI; ~3.2 Mb) and chromosome II (CII; ~0.9 Mb), and five endogenous plasmids [3-6]. It possesses significant metabolic diversity [7-14] and is capable of growing under aerobic, semiaerobic, and photosynthetic growth conditions, while utilizing a wide variety of carbon and nitrogen nutrient sources [15,16]. Therefore, *R. sphaeroides* is an ideal model for the examination and study of gene duplications and their roles in both the evolution of genomic complexity and the metabolic plasticity.

Homologous genes can be classified into two different groups, orthologs or paralogs, depending on the relationship between the genes [17]. Orthologs are homologous genes in different species that originated from a common ancestral gene and they normally retain the same function during the course of evolution. In contrast, paralogs are homologous genes that originated by gene duplication and often contribute to functional innovations that are maintained for adaptation to specific ecological niches. Some gene duplications occur prior to speciation and exist in an ancestral lineage as a pair. These gene duplications are also recognized as paralogs because they are present as a pair in an ancestral genome but were passed on as “co-orthologs” by a speciation event. These types of gene-pairs are referred to as “out-paralogs”, with traditional paralogs (duplication occurring within lineage) being referred to as “in-paralogs” [18,19]. In a previous study, 234 duplicate gene-pairs (paralogs) were identified in *R. sphaeroides* 2.4.1 [20]. Of these, 180 pairs were out-paralogs and 54 were in-paralogs. Duplicated genes often do not evolve to have novel functions, usually becoming silenced [21], but they do have a short opportunity to develop new metabolic capabilities during a brief period of relaxed selection after a duplication event [22]. Additionally, there are several predictions for how duplicated genes are preserved and how they maintain or diverge their functions [23], and some models include neofunctionalization, pseudogenization, subfunctionalization, specialization, and increased protein dosage [19,23].

Microarray expression profiles of *R. sphaeroides* 2.4.1 have revealed that differential expression exists among genes in certain pathways, such as in the components of light harvesting complexes, secondary metabolites, and energy production [24]. The genome of *R. sphaeroides* exhibits genome complexity, an abundance of duplicated genes between its two chromosomes, and varied gene organization (solitary genes and short or long operons) resulting in coordinated, varied metabolic capabilities. The transcription of these complex gene-operons is regulated by transcription regulator(s) under varying environmental conditions, such as oxygen tension and light intensity.

In the current study, four hypotheses were examined. As mentioned above, out-paralogs have vertically descended from an earlier common ancestor as “co-orthologs” while in-paralogs have originated within *R. sphaeroides.* Therefore, the first hypothesis was that in-paralogs have experienced varying levels of selective constraints. Second, it was expected that gene paralogs on different chromosomes (CI and CII) have experienced different selective pressures, since CII sequences evolve more rapidly than the CI sequences in *R. sphaeroides*[25,26]. Third, since protein size is an indicator of gene complexity in eukaryotes [27], it is hypothesized that gene-pairs encoding larger proteins in complex prokaryotic genomes like *R. sphaeroides* are maintained by stronger selection than gene-pairs encoding smaller proteins. Fourth, since transcription of genes is controlled primarily by the interaction of transcription factors (inducers or repressors) and regulatory sequences within the promoter region rather than coding sequences that determine protein structure, it is expected that the differences in gene expression among gene-pairs will not be correlated with nucleotide substitution rate measures in *R. sphaeroides*.

In the current study, the nonsynonymous and synonymous substitution rates (i.e., *K*_*a*_ and *K*_*s*_, respectively) were computed for all 234 duplicated gene-pairs in *R. sphaeroides*. Since out-paralogs and old in-paralogs show saturated level of synonymous substitution rates, selective constraint was measured only for those duplicated gene-pairs, which had *K*_*s*_ values lower than 1.1, so as to provide a reliable estimate of selection. Modes of selection on in-paralogs were also examined and the results were discussed. In addition, microarray expression patterns of duplicate gene-pairs were examined and relationships with the structural constraints measures were investigated. The results of these analyses provide target genes for detailed molecular and biochemical characterization for duplicated genes in the *R. sphaeroides* genome.

## Results

### Variability of *K*_*a*_ and *K*_*s*_ for homologous gene-pairs

**Figure 1:**
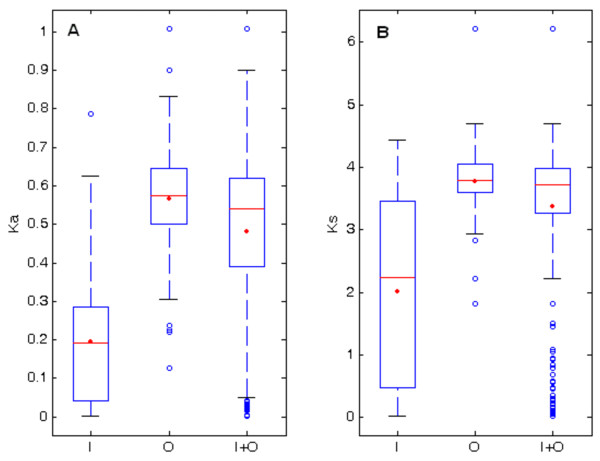
**Whisker box plots detailing the variation in *K***_***a ***_**and *K***_***s ***_**for in-paralogs (I), out-paralogs (O), and all paralogs (I + O).** The distributions of *K*_*a*_ (panel **A)**, and *K*_*s*_ (panel **B)** are shown as box plots with the box representing the interquartile range (25%–75%), the thick solid horizontal line indicating the median, the dot indicating the mean, and the circles indicating the outliers. The plots illustrate and confirm that in-paralogs and out-paralogs possess different distributions of *K*_*a *_and *K*_*s *_*values*.

The *K*_*a*_ and *K*_*s*_ values for in-paralogs, out-paralogs, and all combined paralogs are summarized in Table 1 and the corresponding summary statistics are depicted in whisker box plots in Figure 1. Kolmogorov-Smirnov distribution tests determined that in-paralogs and out-paralogs have significantly different distributions of *K*_*a*_ and *K*_*s*_ as shown in Table 1. As shown in Figure 1, except for a few outliers, *K*_*a*_ and *K*_*s*_ values for out-paralogs were more tightly clustered compared with those for in-paralogs. The higher values of averages of *K*_*a*_ and *K*_*s*_ for out-paralogs are simply indicative of the fact that out-paralogs are ancient (originated prior to speciation), therefore they have experienced a longer evolutionary time, while in-paralogs are young gene duplications originated by duplication event in *R. sphaeroides* lineage.

**Table 1:** **Summary of structural constraint values for paralogs in *R. Sphaeroides***

	**Nonsynonymous substitution rate (*K***_***a***_**)**				**Synonymous substitution rate (*K***_***s***_**)**			
	Range	Average	S.D.	KS Test (P)	Range	Average	S.D.	KS Test (P)
**In-Paralogs**	0.002–0.788	0.195	0.174	1.25e–012	0.028–4.442	2.010	1.511	3.92e–013
**Out-Paralogs**	0.127–1.008	0.566	0.122	4.32e–058	1.825–6.210	3.777	0.452	1.65e–155
**All Paralogs**	0.002–1.008	0.480	0.207	2.84e–013	0.028–6.210	3.369	1.110	2.63e–157

**Figure 2:**
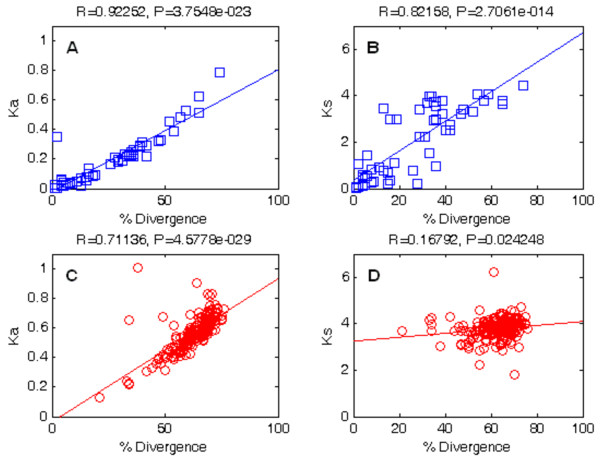
**Correlation between rate of nucleotide substitution (*K***_***a ***_and ***K***_***s***_**) and percentage amino acid divergence.** In-paralogs are denoted as squares, and out-paralogs are denoted as circles. Correlations between *K*_*a*_ and amino acid divergence in in-paralogs and out-paralogs are shown in panel **A** and panel **C**, respectively. Also, correlations between *K*_*s*_ and amino acid divergence in in-paralogs and in out-paralogs are shown in panel **B** and panel **D**, respectively.

Figure 2 displays correlations between amino acid divergence and nonsynonymous or synonymous substitution rates for both in-paralogs and out-paralogs. Statistically significant correlations were found between *K*_*a*_ and amino acid divergence in in-paralogs (R = 0.923, *p* = 3.75E-23) and between *K*_*a*_ and amino acid divergence in out-paralogs (R = 0.711, *p* = 4.58E-29), as shown in panel A and panel C, respectively. Seemingly, a significant correlation was found between *K*_*s*_ and amino acid divergence in in-paralogs as shown in panel B (R = 0.822, *p* = 2.71E-14), while no correlation was found between *K*_*s*_ and amino acid divergence in out-paralogs as shown in panel D (R = 0.168, *p* = 2.42E-2). It is obvious that protein divergence and nucleotide substitution rates are correlated, since the nucleotide substitution rate is the measure of the genetic divergence. However, the strength of correlation is weaker in out-paralogs and thus is indicative of the saturation of nucleotide substitutions as these gene duplications were passed down by speciation.

### Structural constraints operating on duplicated genes

**Figure 3:**
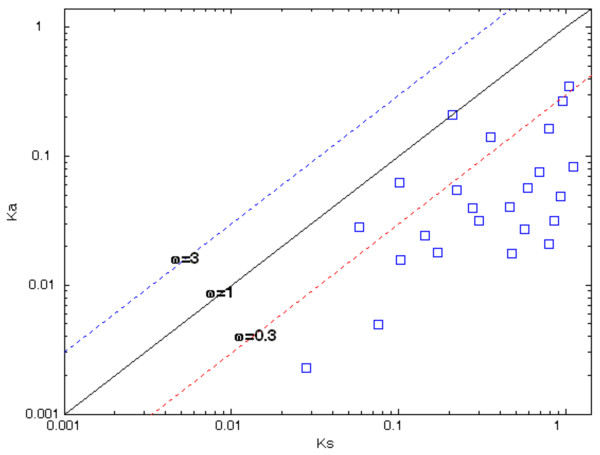
**Pattern of selection on 24 in-paralogs using γ-MYN method.** The graph and axes are shown on a logarithmic scale. The diagonal lines from bottom to top represent *ω* = 3, *ω* = 1, and *ω* = 0.3 to provide a visual measure for the levels of selection operating on the gene pairs. The selective constraint was calculated on only the selected 24 in-paralogs, which exhibit *K*_*s*_ values < 1.1. The majority of the in-paralogs lie on or under the line of *ω* = 0.3 indicating that they are under purifying or negative selection.

As mentioned before, the selective constraint (*ω*) is the ratio of non-synonymous substitution rate (*K*_*a*_) to synonymous substitution rate (*K*_*s*_) and it is therefore used as an indicator of the selective pressure acting upon a gene-pair. It is known from previous studies that the estimation of selective constraint is not reliable for values of *K*_*s*_ > 1 because of the saturation artifact of synonymous sites for the old paralogs [28,29]. As shown in Additional file 1: Table A1, all 180 out-paralog gene-pairs and 30 of the total 54 in-paralog gene-pairs revealed values of *K*_*s*_ > 1, and therefore the estimated *ω* values for these 210 paralog gene-pairs may not be reliable for predicting the mode of selection constraint. A majority of gene-pairs are located within and between chromosomes, while 23 and 15 gene-pairs were placed between chromosome and plasmid and between plasmids, respectively. Although it remains to be determined to what extent genes were horizontally transferred in the *R. sphaeroides* genome, it is likely that gene homologs distributed either between plasmids or between a chromosome and a plasmid are possibly acquired by horizontal gene transfer (HGT) event. Table 2 describes the various parameters of evolutionary constraints as well as gene functions of the remaining 24 in-paralog gene-pairs, which exhibit *K*_*s*_ < 1.1, and therefore these in-paralog gene-pairs will be reliable indicators of the selective constraints in *R. sphaeroides* genome as shown in Figure 3. Whereas most methods, which measure the selective constraints, identify negative selection at *ω* < 1, neutal selection at *ω* = 1, and positive selection at *ω* > 1, γ-MYN (Modified Yang-Nielsen) method predicts negative selection at *ω* ≤ 0.3, neutral selection at 0.3 < *ω* < 3, and positive selection at *ω* ≥ 3 [28]. The relationships between the *K*_*a*_ and *K*_*s*_ of the 24 in-paralog gene-pairs using γ-MYN are shown in Figure 3, which also demonstrates that 19 in-paralog gene-pairs are under purifying selection (*ω* ≤ 0.3) and only 5 gene pairs operate under neutral selection (0.3 < *ω* < 1), according to γ-MYN method. 

**Table 2:** **Duplicate gene-pairs in *R. sphaeroides *with selective constraint (ω) < 1 (ordered by ω)**

^**a**^Gene 1	^a^Gene 2	**Function**	^b^Location	^c^Length	^d^Divergence	^**e**^***Ka***	^e^***Ks***	^e^ω	^f^Correlation	^g^Cosine
RSP_1647	RSP_3650	Protease	CI/CII	312	13	0.0207	0.7894	0.0262	0.7507	0.9805
RSP_6194	RSP_6200	Hypothetical	CII/CII	140	4	0.0174	0.4721	0.0369	N/A	N/A
RSP_3624	RSP_3792	Hypothetical	CII/CII	238	8	0.0318	0.8434	0.0377	0.7706	0.9452
RSP_2482	RSP_4189	Receptor	PC/PD	294	6	0.0270	0.5608	0.0481	0.9162	0.9250
RSP_2064	RSP_6012	Hypothetical	CI/CI	126	11	0.0485	0.9247	0.0525	N/A	N/A
RSP_4252	RSP_3907	Hypothetical	PD/PA	163	2	0.0050	0.0757	0.0658	N/A	N/A
RSP_1653	RSP_6190	Hypothetical	CI/CII	208	18	0.0821	1.0949	0.0750	N/A	N/A
RSP_3908	RSP_4251	Hypothetical	PA/PD	185	1	0.0023	0.0279	0.0811	N/A	N/A
RSP_1638	RSP_2062	Hypothetical	CI/CI	126	5	0.0406	0.4615	0.0881	0.7369	0.7604
RSP_1645	RSP_3652	Phage‐related	CI/CII	419	4	0.0565	0.5853	0.0965	0.9933	0.9694
RSP_2063	RSP_1639	Hypothetical	CI/CI	147	9	0.0317	0.3017	0.1051	0.9995	0.9700
RSP_3627	RSP_3784	Protease	CII/CII	448	5	0.0180	0.1697	0.1061	N/A	N/A
RSP_7390	RSP_3896	Transposase	PD/PA	413	15	0.0759	0.6951	0.1091	N/A	N/A
RSP_1956	RSP_6196	Hypothetical	CI/CII	124	8	0.0396	0.2751	0.1439	N/A	N/A
RSP_4138	RSP_3902	Hypothetical	PD/PA	265	4	0.0156	0.1028	0.1513	N/A	N/A
RSP_1951	RSP_3622	Hypothetical	CI/CII	116	4	0.0242	0.1428	0.1692	0.2186	0.7307
RSP_4178	RSP_3012	Transposase	PD/CII	119	26	0.1629	0.7886	0.2066	0.5797	0.9629
RSP_2061	RSP_1637	Hypothetical	CI/CI	70	15	0.0548	0.2219	0.2467	0.1230	0.1230
RSP_6035	RSP_3772	Hypothetical	CI/CII	113	36	0.2654	0.9499	0.2794	N/A	N/A
RSP_3904	RSP_7352	Conjugation	PA/PE	638	2	0.3479	1.0456	0.3327	N/A	N/A
RSP_3628	RSP_3786	Hypothetical	CII/CII	106	16	0.1400	0.3513	0.3985	0.1197	0.8483
RSP_1966	RSP_3007	Transposase	CI/CII	242	1	0.0279	0.0577	0.4842	0.9688	0.9963
RSP_3894	RSP_3978	Transport	PA/PB	276	4	0.0620	0.1005	0.6171	0.0315	0.9396
RSP_1955	RSP_3647	Hypothetical	CI/CII	219	28	0.2077	0.2090	0.9937	0.7644	0.8779

^a^gene name as shown in *R. sphaeroides* 2.4.1 annotation at NCBI.

^b^location: chromosome I (CI), chromosome II (CII), or plasmids (PA,PB, PC, PD, or PE).

^c^average length of the duplicate genes.

^d^percentage of amino acid divergence between protein homologs.

^e^evolutionary constraint parameters: nonsynonymous substitution rate (*K*_*a*_), synonymous substitution rate (*K*_*s*_), and *K*_*a*_/ *K*_*s*_ (ω).

^f^Pearson’s correlation.

^g^cosine similarity.

Note that “N/A” is given for the pair, whose corresponding gene expression does not exist in the microarray.

**Figure 4:**
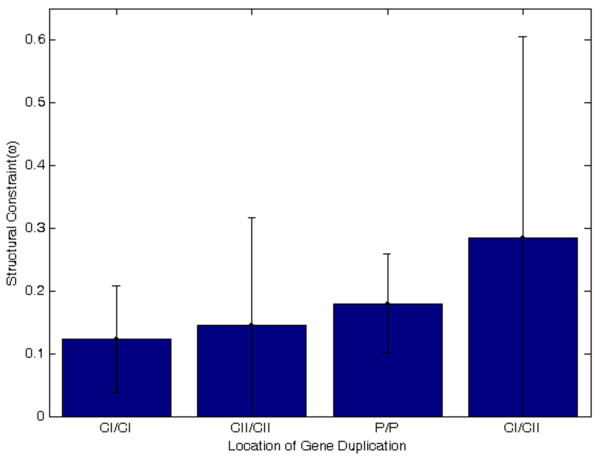
**Average *ω *values of in-paralog gene-pairs contained within CI, within CII, within plasmids, and between CI and CII.** The distribution of the average values of *ω* suggests that inparalog gene-pairs, which are located on different chromosomes, experience different selection pressures.

The distribution of average *ω* values for in-paralog gene-pairs located within and between chromosomes is shown in Figure 4. For the 4 gene duplications that exist within CI, the average *ω* value was 0.123 ± 0.085. For the 4 gene duplications that exist within CII had an average *ω* value of 0.145 ± 0.172. The average ω value of the 7 duplications between plasmids was 0.180 ± 0.079. A total of 8 duplications were distributed between CI and CII and had an average *ω* value of 0.284 ± 0.321. The average *ω* values of in-paralogs distributed within and between replicons are not significantly different.

**Figure 5:**
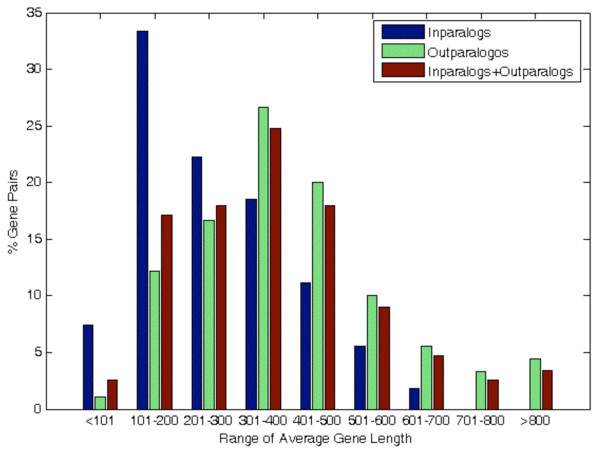
**Frequency of gene-pairs of different lengths for in-paralogs, out-paralogs, and all paralogs.** The frequency of in-paralogs and out-paralogs dramatically differs over varying ranges of average gene lengths.

Figure 5 shows the frequency (%) of paralogs of different gene lengths and illustrates that the in-paralogs are observed more frequently in short gene lengths (<100, 101–200, and 201–300 base pairs), while the out-paralogs were present more frequently in long gene lengths (301–400, 401–500, 501–600, and 601–700 base pairs). Specifically, 18 of the total 54 in-paralogs (~33%) and 22 of the total 180 out-paralogs (~12%) were present in the range of 101–200 base pairs. In contrast, no in-paralog was found in long gene length > 700 base pairs. Kolmogorov-Smirnov test (*p* = 0.958) on these values also indicated that relative frequencies of gene pairs in in-paralogs and those in out-paralogs are similarly distributed among all the classes of average gene lengths.

The average *ω* values of protein-pairs grouped by length are roughly similar (data is not shown), therefore protein length was not correlated with structural constraint (*ω*). Accordingly, it may indicate that genes with longer length appear to be subject to similar levels of purifying selection as those with shorter length.

### Different gene expression patterns among duplicated genes

**Figure 6:**
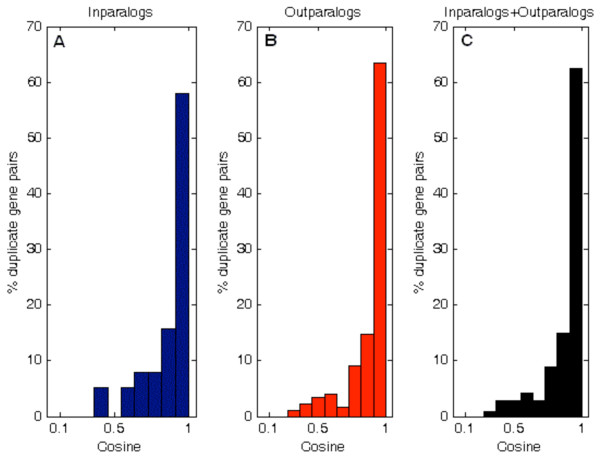
**Similarity in expression patterns between duplicated genes.** The plots represent in-paralogs (panel **A)**, out-paralogs (panel **B)**, and all paralogs (panel **C)**, respectively. As a reference, cosine value equal to one (i.e., 1) represents identical patterns of expression across all growth conditions for a given gene-pair. As shown, in-paralogs and out-paralogs possess similar distributions of cosine values where the vast majority of gene-pairs possess cosine > 0.5.

Out-paralogs and in-paralogs possess similar distributions of cosine values as shown in Figure 6. Of the total paralogs, 133 (~62%) gene-pairs possessed cosine ≥ 0.9, while only 14 (~7%) gene-pairs had cosine ≤ 0.5. Although microarray experiment utilized probes that minimize the cross-hybridization, the direct consequences of the cross-hybridization between genes with high sequence similarity (specifically in-paralogs) may have been underestimated.

**Figure 7:**
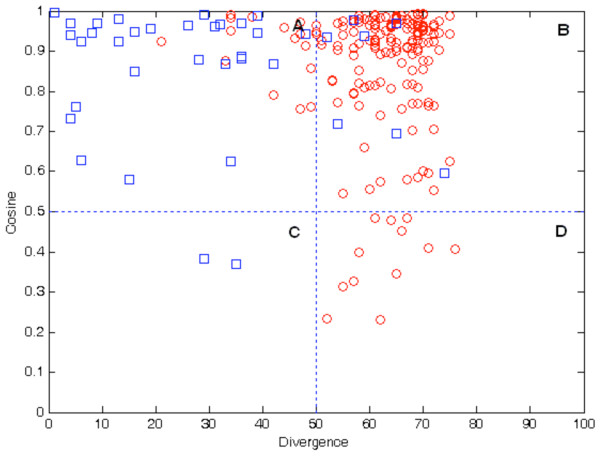
**Representation of in-paralogs (circles) and out-paralogs (squares) in four different categories of expression patterns.(A)** High cosine (>0.5) with low divergence (≤50%), **(B)** high cosine with high divergence (>50%), **(C)** low cosine (≤0.5) with low divergence, and **(D)** low cosine with high divergence. The majority of out-paralogs are clustered in group B (high divergence with high cosine).

**Figure 8:**
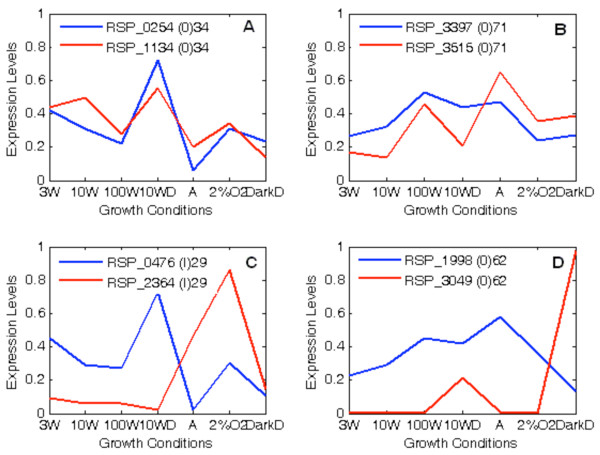
**Four example patterns of normalized gene expression.** The darker line represents one copy within a pair and the lighter line represents the other copy. Gene identification numbers (RSP), homolog type (O or I), and percent divergence of each copy in the pair are given at the top of each graph. Each graph is an example from the previously defined four expression classifications: **(A)** High cosine (>0.5) with low divergence (≤50%), **(B)** high cosine with high divergence (>50%), **(C)** low cosine (≤0.5) with low divergence, and **(D)** low cosine with high divergence. It is evident that gene-pairs with high cosine have similar expression patterns while those with low cosine have dissimilar expression patterns.

Expression patterns of duplicated genes were classified into four groups as illustrated in Figure 7: (A) high cosine (>0.5) with low divergence (≤50%), (B) high cosine with high divergence (>50%), (C) low cosine (≤0.5) with low divergence, and (D) low cosine with high divergence. A representative of gene expression pattern of each group was shown in Figure 8, which will be discussed further. Although duplicate genes were present in each of the four categories of expression patterns, many out-paralog gene-pairs were present in the group of high divergence with high cosine (group B). There are 18 (~8%) out-paralogs and 29 (~14%) in-paralogs in group A, 145 (~68%) out-paralogs and seven (~3%) in-paralogs in group B, only two (~1%) in-paralogs in group C, and 12 (~6%) out-paralogs in group D. Out-paralogs generally had higher divergence than in-paralogs with divergence ranging from 21% to 76%, while cosine values ranged from 0.231 to 0.993. In-paralogs generally possessed higher cosine values with a range from 0.370 to 0.996, while divergence ranged from 1% to 74%. Two-sample Kolmogorov-Smirnov test (*p* = 0.832) indicated that cosine values of in-paralogs and out-paralogs are similarly distributed.

Correlations between *K*_*a*_ and expression divergence and between *K*_*s*_ and expression divergence for in-paralogs are shown in Additional file 2: Figure A1. Both correlations were not strong as follows: correlation between *K*_*a*_ and expression divergence (R = 0.359, *p* = 2.70E-2) and correlation between *K*_*s*_ and expression divergence (R = 0.441, *p* = 5.61E-3).

**Figure 9:**
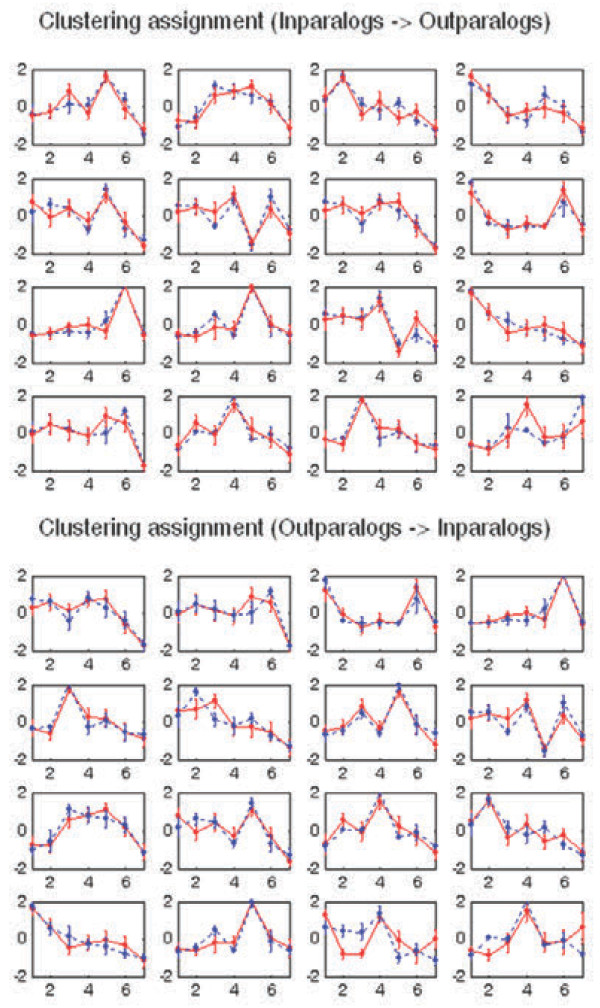
**Representation of similarity in expression pattern among paralogs.** There are 16 dominant gene expression patterns in both in-paralogs and out-paralogs and these paralogs have high cosine expression similarity at the individual gene to gene and cluster to cluster levels, indicating that individual gene expression patterns in out-paralogs are similar to those in in-paralogs.

The average cosine values of individual gene similarities are 0.913 ± 0.069 and 0.952 ± 0.037 for out-paralogs to in-paralogs gene search and for in-paralogs to out-paralogs gene search, respectively. These high cosine values indicate that almost every gene in out-paralogs has a very similar gene expression in in-paralogs, and vice versa. The 16 latent expression patterns revealed by *k*-means with hierarchical agglomerative clustering (HAC) initialization were illustrated in Figure 9 (in-paralog to out-paralog cluster search) and Figure 9 (out-paralog to in-paralog cluster search), where 12 expression patterns were symmetrically best matched between out-paralogs and in-paralogs. Although the remaining four expression patterns are not symmetrically best matched, the expression patterns remain similar. The average cosine values of the matched cluster similarities are 0.855 ± 0.133 (for out-paralog to in-paralog cluster search) and 0.883 ± 0.091 (for in-paralog to out-paralog cluster search).

## Discussion

### Duplicated genes are maintained by purifying selection

In the current study, four specific hypotheses were examined. The first hypothesis was that two types of paralogs (namely in-paralogs and out-paralogs) in *R. sphaeroides* genome will have varying levels of evolutionary constraints (*K*_*a*_ and *K*_*s*_). In particular, wider degree of variability in evolutionary constraints was observed in out-paralogs than in in-paralogs. This supports the previous finding that most ancient gene duplications in *R. sphaeroides* saturate synonymous substitutions (*K*_*s*_), and the corresponding selective constraint (ω) would decrease with increasing time as found in several bacteria including *Escherichia coli* and *Staphylococcus aureus*[30,31]. Furthermore, duplicated genes in *Caenorhabditis elegans*, *Saccharomyces cerevisiae*, *Arabidopsis thaliana*, *Drosophila melanogaster,* bacteria, and mammals have all evolved under purifying selection [32]. Since older duplications (out-paralogs and many old in-paralogs) are inherent to have *K*_*s*_ > 1, the measure of selective constraint may not be reliable; however, majority of young in-paralogs showed that these gene-pairs are maintained under purifying selection.

Duplicated genes tend to experience a brief period of relaxed selection soon after the duplication event, thereby leaving opportunities for metabolic innovation [21,33-35]. Some in-paralogs have not reached their maximum level of non-synonymous changes and so gene copies are allowed to harbor mutations, which may lead to expansion of gene functions. Relaxation of constraints can be the cause of an adaptive change that can alter protein function, so positive selection may occur at *ω* < 1 [36].

Gene-pairs were determined to be relatively recent in *R. sphaeroides* genome, if they possessed *K*_*s*_ < 1.1 and divergence < 50% as shown in Table 2. If functional constraints are actually relaxed immediately after duplication, genes with these characteristics should also have elevated *ω* values [37]. The average *ω* value for the most recent gene duplications within *R. sphaeroides* 2.4.1 was high and thus it indicates that relaxed selection may be acting on these gene-pairs, allowing them to gain metabolic novelties. Of the 24 in-paralogs listed in Table 2, 15 in-paralogs code for hypothetical proteins and the others code for functions like transport, transposase, protease, conjugation, receptor, and phage-related protein function. As all of these in-paralogs originated in the *R. sphaeroides* lineage, varied gene function within a gene-pair is consistent with previous findings that in-paralogs experience accelerated evolution and can accumulate more amino acid substitutions [32]. In a similar vein, as analyzed in *Escherichia coli*, *Helicobacter pylori*, and *Neisseria meningitides*, gene duplications evolve at faster rates compared to unique single-copy genes but this finding was not consistent in *Chlamydophila pneumoniae*[37]. These findings also confirm that duplicated genes are often involved in less critical functions and that these may be responsible for strain-specific differences [37].

### Similar level of evolutionary constraints on duplicate genes located on CI and CII

In *R. sphaeroides* 2.4.1, CII has been shown to be rapidly evolving [25,26]. For this reason, the second hypothesis was that gene paralogs on different replicons might have experienced different selective pressures within *R. sphaeroides* and specifically that paralogs between CI and CII might have experienced different levels of selection from those contained strictly within CI, within CII, or within plasmids. Our result revealed that the mean *ω* value of in-paralogs distributed between CI and CII (*ω* = 0.284 ± 0.321) is higher than the mean *ω* value of in-paralogs located within CI (ω = 0.123 ± 0.085*)* and also within CII (ω = 0.145 ± 0.172), but the difference in *ω* is not statistically significant. Thus, the selective constraint is not the primary force for the rapid divergence of CII in *R. sphaeroides*. These findings are consistent with the previous observation that 28 gene orthologs within CI and CII among four strains of *R. sphaeroides* are maintained under purifying selection [20].

In an analysis of 28 gene-pairs common among four strains of *R. sphaeroides* (2.4.1, ATCC17025, ATCC17029, and KD131), purifying selection (ω ≤ 0.3) was detected under the MYN model [20], indicating that these gene-pairs have significantly conserved their functions, and have not possibly evolved via convergent evolution. Some duplicated genes in *R. sphaeroides* 2.4.1 might possess evidences of being subfunctionalized or specialized for similar functions, but other pairs may also be maintained through neofunctionalization. As a note, very few genes in genomes generally possess significantly high ω values [38]. This is partly due to the fact that genomic streamlining can lead to limited size, and microbial genomes lack a permissive environment for expansion of nun-functional DNA due to different population genetic environments [39].

Different structural constraints exist for genes with different functions. Essential genes are highly conserved within bacterial genomes [40,41] and duplicated genes in *R. sphaeroides* follow this trend. Herein then, one significant explanation for the differences in structural constraints across *R. sphaeroides* replicons could lie in the distribution of essential genes across its replicons. Generally, for bacteria with two chromosomes, there is one large primary chromosome (CI) that contains a great deal of essential genes, while the accessory chromosome (CII) contains a sizeable percentage of nonessential genes, coding for hypothetical proteins of unknown function [42]. One issue that arises, however, is whether the presence of nonessential genes contributes to more variability of selection pressures on CII or weaker selection pressures on CII allow for the presence of nonessential genes to thrive and diverge. Although it is not immediately clear which predominates, it is probably a combination of both situations. As hypothesized before [26], it is likely then that relaxed structural constraints on the CII of bacteria significantly contributes to their increased evolution and divergence compared to CI, although the findings of the current study may stand in contrast to that idea.

### Protein size does not influence selective constraints on coding sequence evolution

Although protein size is an indicator of gene complexity [27,43,44], *K*_*a*_, *K*_*s*_, and *ω* were not correlated with gene length in *R. sphaeroides* 2.4.1. In accord, the third hypothesis, which postulated that other gene-pairs coding for larger proteins would be maintained by stronger selection than other gene-pairs coding for smaller proteins, was not substantiated. However, owing to the limited number of duplicate genes that code proteins of varying lengths, the hypothesis remains to be tested on a larger data set. As a note, the stronger selection pressure exists on larger prokaryotic genomes compared to smaller ones [29]. However, in eukaryotes, bigger proteins are likely more important compared to in prokaryotes [43], therefore longer and more complex genes are often selected to be duplicated in eukaryotes [27].

### Gene expression patterns and structural constraints

Duplicated genes display a variety of expression patterns across seven growth conditions. Most in-paralogs show high cosine values, which suggests that these gene-pairs are similarly expressed, while the majority of out-paralogs possess a wide range of cosine values, which is indicative of their varied expression patterns. Varied expression patterns among out-paralogs suggest that out-paralog gene pairs evolved either to produce more proteins required by specific metabolic functions [45] or to specialize new gene functions. The present results are in contrast to previous findings that gene duplications solely serve to increase protein dosage [46] and our findings also suggest that gene duplication may play an important role in specializing new functions as a variety of expression patterns were observed for many of these duplicate gene pairs [47-50].

As illustrated in Figure 9, there exist 16 dominant or discriminating expression patterns in both in-paralogs and out-paralogs. These paralogs have high cosine expression similarity at gene–to-gene and cluster-to-cluster levels, indicating that individual gene patterns in out-paralogs are similar to those in in-paralogs. Furthermore, all the latent major patterns observed in out-paralogs were preserved in in-paralogs with a very little variation. No distinct expression patterns in either out-paralogs or in-paralogs were observed.

Figure 8 presents four examples of expression patterns for gene-pairs corresponding to each of the four previously defined groups (A, B, C, and D). It is immediately evident that gene-pairs with high cosine (>0.5) exhibit fairly similar expression patterns across all growth conditions while those with low cosine (≤0.5) exhibit dissimilar expression patterns. More specifically, as shown in Figure 8C and 8D, there is a divergent level of expression for the low cosine gene-pairs with the genes in each pair peaking at different conditions. In addition, protein divergence does not seem to be relevant to the expression similarity patterns as shown in Figure 8A and 8B. Additionally, although gene expression in out-paralogs does not appear to be related to structural constraints, *K*_*a*_ and *K*_*s*_ were weakly correlated (*p* < 0.05) with expression divergence in in-paralogs. As in-paralogs are relatively newer duplications compared to out-paralogs, these findings could suggest that in a gene duplication event, expression divergence is first partially due to structural constraints acting upon the gene-pair. However, as time increases, such structural constraints begin to matter less and other regulatory factors come into play. The fourth hypothesis submits that gene expression is not correlated with *K*_*a*_ and *K*_*s*_ because changes in regulatory elements will have a greater impact on the level of expression than changes in the coding sequence. This hypothesis is only partially supported, as there is a lack of significant correlations between those factors in out-paralogs but weak correlations exist in in-paralogs.

Therefore, in out-paralogs and possibly old in-paralogs, it seems then that other factors might be at work in contributing to expression divergence. One such factor is the promoter region of these genes. For instance, during a period of relaxed selection, some regions of a gene may be maintained or conserved, while others are altered [38] and interact differently with transcriptional regulators. As such, it is possible that promoter sequences in some duplicated genes in *R. sphaeroides* have been selectively conserved after duplication but coding sequences have diverged so that gene-pair functions can differ but promoters can respond to similar environmental or physiological cues. On the other hand, if a similar gene structure or coding region is maintained but promoters have diverged, a gene-pair can be specialized for functions in a particular environmental condition. In prokaryotes, paralogs have been associated with advantageous changes or mutations such as in methyltransferase which are adaptive to the environment [51]. Similarly, it has been proposed that gene duplications are useful for fluctuations in the environment so that each copy can perform the same function under different conditions [52]. For instance, in *R. sphaeroides,* duplicated *cbb* genes participate in carbon fixation pathways, but different forms of the enzyme function under different levels of oxygen tension [20]. Such an understanding is further substantiated by data shown in Figure 8. For instance, as mentioned before, Figure 8C and 8D present gene-pairs with low cosine. The genes in Figure 8C are RSP_0476 and RSP_2364 and they code for L-fuculose-phosphate aldolase (Class I) and L-fuculose phosphate aldolase (Class II ), respectively. The graph demonstrates that these two genes are expressed differently under the growth conditions, although their amino acid divergence is fairly low (29%). Likewise, in Figure 8D, the genes RSP_1998 and RSP_3049 code for molybdopterin molybdochelatase and molybdenum cofactor biosynthesis protein, respectively. These genes follow a similar pattern as those in Figure 8C, even though their amino acid divergence is high (62%). Therefore, it is more likely that diverged promoter sequences for these sets of genes have resulted in the differential gene-pair expression.

As such, in the cases in which differential expression exists between genes within a duplicate pair, it is possible that the promoter regions are not conserved within the pair. In a duplication event, promoter sequences may or may not have been duplicated with the coding region or the duplicated promoter regions may have diverged, if the duplicate copy is located close to another operon and is now controlled by a different, new promoter. Either case would therefore result in different patterns of expression between the gene copies. Additionally, paralogs have been shown to diverge with respect to regulatory control rather than biochemical function of the associated protein, possibly leading to differences in expression patterns [53]. Analysis of promoter regions of duplicate genes is under investigation, and therefore the result is not currently available.

The current study demonstrated that duplicated genes have distinct patterns of gene expression that may be related to the function and maintenance of each gene copy in the genome of *R. sphaeroides*. Through analysis of structural and functional constraints on duplicated genes in *R. sphaeroides*, many paralogs were maintained under negative selection, indicating that their functions are constrained. Because most gene duplications are out-paralogs, the period in which they experienced relaxed selection may not be detectable. Moreover, duplicated genes are maintained with varying levels of amino acid divergence, and ~62% have nearly identical expression across seven different growth conditions. However, these results also mean that gene copies are allowed to diverge and if a new adaptive function is acquired by mutation, the new diverged gene may become subject to negative selection in order to maintain that function.

Furthermore, the results of this study have set the cornerstone for detailed molecular analysis of duplicated genes in prokaryotes and specifically in *R. sphaeroides*. Future studies may incorporate techniques such as promoter-swapping and gene knockout to examine important gene regulation mechanisms and regulatory circuits that facilitate functional innovations in *R. sphaeroides* and other prokaryotic organisms. In addition, future studies should examine gene-gene interaction networks with respect to gene duplication as well as in relation to the growth environments. For instance, one recent study suggests that the role of gene duplications are overstated in the evolution of metabolic functions and protein families in that duplicated genes are primarily important in gene dosage but horizontal gene transfer is the primary method of acquiring of new and evolved functions in organisms [46]. As such, examination of not only major evolutionary forces, such as horizontal gene transfer and gene duplication, but also the interplay of these forces is essential to understand prokaryotic genome complexity and the evolution of new gene functions.

## Conclusions

Aside the variability in structural-functional constraints (*K*_*s*_, *K*_*a*_, and *ω*) among duplicate gene pairs, a majority of out-paralog and in-paralog gene-pairs in *R. sphaeroides* are maintained under negative evolutionary pressure (purifying selection), and the finding is consistent with the results previously reported on other species. Only a small percentage of paralogs evolves into novel metabolic functions in *R. sphaeroides*. Two chromosomes (CI and CII) revealed a very similar level of evolutionary constraint and therefore the selective constraint on the duplicated gene-pairs is not the major force for the rapid genetic divergence of CII. In addition, expression patterns of duplicated gene-pairs suggest that a majority of duplicated genes in *R. sphaeroides* are similarly expressed over several growth conditions, however the level of similarity (cosine values) varies among duplicated gene-pairs. Only 14 gene-pairs have very divergent gene expression patterns. This study concludes that gene duplication not only means to innovate gene functions, but also contributes towards increasing protein dosages, which help this organism to adapt to its environment.

## Methods

### Selective constraint analysis

All 234 gene-pairs identified in a previous study [20] were used for analysis. To analyze the selective constraints operating on the duplicate genes, automated software was employed in a PERL script and is available upon request. The program performs the following steps: first, nucleic acid sequences in FASTA format are translated to amino acids using the standard codon table; second, the translated protein sequences are stored in a separate file for each pair; third, the protein sequences for each duplicate protein-pair are then aligned using MUSCLE [54]; fourth, the aligned protein sequences are back-translated into the original corresponding DNA sequences using PAL2NAL [55]; fifth, each pair of DNA sequences is then subjected to KaKs_Calculator for analysis [56]. KaKs_Calculator identifies the synonymous (*K*_*s*_) and non-synonymous (*K*_*a*_) nucleotide substitutions between two sequences and then computes the nonsynonymous/synonymous rate ratio (*ω* = *K*_*a*_/*K*_*s*_). It is better to align protein sequences rather than nucleic acid sequences because protein alignments prevent the introduction of frame shifts that may occur due to the incorrect placement of gaps during alignments [57]. KaKs_Calculator implements several estimation models, including the MYN (Modified Yang-Nielsen) method [58,59]. In addition, it includes model selection, model averaging, and γ-distribution, which can be applied to these methods for the estimation of *ω*[60-62]. Plots and regression analysis were generated with MATLAB 7.11. Kolmogorov-Smirnov tests [63] were performed using online software ( http://www.physics.csbsju.edu/stats/KS-test.html). The level of significance for all statistical tests was designated at α = 0.05.

The MYN method adopts the Tamura-Nei (TN) model of substitution [64] at each of the three steps in its calculation [56]. A modified form of MYN, γ-MYN, is based on the assumption that the evolutionary rate at each site follows a mode of γ-distribution because unequal substitution rates affect *K*_*a*_ and *K*_*s*_[28]. This distribution can also be applied to other methods incorporating a γ-distribution shape parameter to suggest that variable substitution rates across sites under negative and positive selection have different effects on the estimation of *ω*[61]. Different mutation models using different evolutionary parameters may produce biased results so it is important to choose the appropriate modeling for a particular sequence. Since the MYN method assumes that different nucleotide positions evolve at the same rate, the addition of the γ-distribution parameter attempts to increase biological realism [28]. Added parameters can cause redundancy, but the γ-MYN method models γ-distribution at the amino acid level, which likely avoids this problem [28].

### Gene expression analysis

The microarray expression data for all genes in *R. sphaeroides* 2.4.1 grown under seven different growth conditions have been published [24]. The seven growth conditions include aerobic, anaerobic, semi-aerobic, dark/dimethyl-sulfoxide (DMSO), and three photosynthetic conditions under different light intensities (3 watts, 10 watts, and 100 watts), where three replicates for each growth condition were presented. It is noted that the probes for the microarray were designed so that under stringent hybridization condition, it produces minimal cross-hybridization. In addition, the results from the microarray experiment were independently verified with previous observations on gene expression performed by northern blot and qPCR analysis [24]. Of the 234 duplicate gene-pairs, the gene expression levels of 213 duplicate gene-pairs were available in the microarray expression data. The three replicates were averaged to form the expression level for each growth condition, resulting in a rectangular real-value data matrix with 426 rows (213 gene-pairs) and seven columns (seven growth conditions). Then, rows (genes) of the gene expression data matrix were normalized by z-score transformation to reduce difference in scale among each gene expression, resulting in mean 0 and variance 1 of every gene expression over the seven growth conditions. The normalized expressions of homologs are clustered with the usual Hierarchical Agglomerative Clustering (HAC) and shown in Additional file 3: Figure A2. From a biological point of view, the relative up- or down-regulation of gene expression is interesting, instead of the absolute amplitude changes. Therefore, z-score transformation has been used to emphasize the relative variation in intensity among genes or samples/conditions in gene expression [65,66].

Pearson’s correlation coefficient (R) was used to quantify the correlation between two gene expression patterns of each duplicate pair and then linear regression analysis was employed to investigate the relationship between R and each of the evolution constraints (*K*_*a*_, *K*_*s*_, or *ω*), where the transformed R(= ln[(1 + R)/(1 - R)]) was used to follow the previously published approach [47] that uses the transformed R to change the scale of R to be appropriate for a linear regression analysis. Linear regression analysis was also used to analyze relationships between expression divergence and each of the evolution constraints. In addition, the cosine similarity [67] metric was used to measure the similarity between two gene expression patterns of each duplicate pair. Each gene is represented in the vector of the seven growth conditions (i.e., in the seven dimensional space) and cosine similarity measures the cosine of the angle formed by the two gene vectors, through which cosine quantifies how closely two gene vectors point in the high dimensional space. If the two genes are similarly expressed, cosine is close to one (i.e., 1), since the angle between the two gene vectors is close to 0 degrees, pointing in the same direction. If they are differently expressed, cosine is close to zero (i.e., 0), since the angle is close to 90 degrees.

In addition, to reveal the latent expression patterns in the microarray data, both HAC with average linkage and *k*-means clustering algorithms were applied to cluster genes into groups of similar or consistent patterns across the growth conditions. As a note, one minus the cosine was used as the distance metric for both the algorithms. HAC is utilized as an initialization for *k*-means, which resolves the initialization problem and thus results in a deterministic clustering with *k*-means. HAC has been successfully applied as an initialization for other clustering algorithms [66,68-70].

To identify conserved patterns between in-paralogs and out-paralogs as well as paralog-specific co-expression patterns either in in-paralogs or in out-paralogs, a very simple and straightforward method was developed as follows. First, the clustering for in-paralogs and the clustering for out-paralogs were obtained separately using *k*-means with HAC initialization. Then, each cluster (as a query) in the clustering of in-paralogs was matched to the closest cluster (as a library) in the clustering of paralogs and vice versa, where cosine similarity was also used to measure the closeness between query and library clusters. This process resembles the BLAST search and results in two pairs of the best matches, one for in-paralogs to out-paralogs match and the other for out-paralogs to in-paralogs match. The similarity of all the matched cluster pairs was quantified using the average cosine similarity, the conserved patterns (i.e., the best matched clusters) were visualized, and the biological functions of the conserved patterns were discussed.

## Abbreviations

*K*_*s*_: ynonymous substitution rate; *K*_*a*_: Non-synonymous substitution rate; *ω*: Selective constraint; MYN: Modified Yang-Nielsen method; HAC: Hierarchical agglomerative clustering; ABC: ATP binding cassette; HGT: Horizontal gene transfer DMSO: Dar/demethyl-sulfoxide.

## Competing interests

The authors declare that they have no competing interests.

## Authors’ contributions

All authors (AP, AB, HC, MC) have substantially contributed to the study and manuscript. All authors were integral for the collection and analysis of the data and the formation of the manuscript. All authors approve of the final manuscript.

## Availability of supporting data

The supplementary data describing the results in this article are included as additional files.

## Supplementary Material

Additional file 1 **Table A1.** Information of all the 234 duplicate gene-pairs in *R. sphaeroides.* Click here for file

Additional file 2 ** Figure A1.** Relationship between normalized correlation values and structural constraints on duplicated genes in *R. sphaeroides*. In-paralogs are shown in blue squares and out-paralogs are shown in red circles: (A) *K*_*a*_ of in-paralogs, (B) *K*_*s*_ of in-paralogs, (C) *K*_*a*_ of out-paralogs, and (D) *K*_*s*_ of out-paralogs. Click here for file

Additional file 3 ** Figure A2.** Hierarchical clustering of the normalized expressions of homologs in R. sphaeroides 2.4.1. Each column represents the following growth condition: (1) 3W, (2) 10W, (3) 100W, (4) 10W DMSO, (5) Aerobic, (6) 2% Oxygen, and (7) Dark DMSO. Three replications of each growth condition are averaged and the averaged expression levels are normalized by z-score transformation before clustering with hierarchical clustering. Green represents low levels of expression while red represents high levels of expression. Click here for file
