# Supplementary material for: Evolutionary constraints and expression analysis of gene duplications in Rhodobacter sphaeroides 2.4.1
Source: BMC Res Notes. 2012 Apr 25;5:192. doi: 10.1186/1756-0500-5-192 (PMC3494609; doi:10.1186/1756-0500-5-192)
Supplement: Additional file 1 — Table A1. Information of all the 234 duplicate gene-pairs in R. sphaeroides. [file 1756-0500-5-192-S1.pdf]

Table A1 Information of all the 234 duplicate gene-pairs in *R. sphaeroides*

NO.	[a]Gene 1	[a]Gene 2	[b]Location	[c]Paralog	[d]Length	[e]Divergence	[f]K_a	[f]K_s	[g]Correlation	[h]Cosine
**1**	RSP_0036	RSP_1325	CI/CI	O	181	67	0.619718	3.26571	-0.24183	0.483044
**2**	RSP_0047	RSP_1588	CI/CI	O	367	58	0.502641	3.95110	0.95684	0.972713
**3**	RSP_0054	RSP_2220	CI/CI	O	349	76	0.729505	3.76165	-0.31253	0.405763
**4**	RSP_0056	RSP_1332	CI/CI	O	439	61	0.600079	6.20966	-0.08762	0.483235
**5**	RSP_0061	RSP_1310	CI/CI	O	93	65	0.597111	2.82532	-0.23828	0.344105
**6**	RSP_0064	RSP_1328	CI/CI	O	88	55	0.540962	2.83228	-0.39388	0.314538
**7**	RSP_0065	RSP_1321	CI/CI	O	265	66	0.673754	3.85787	-0.20476	0.452827
**8**	RSP_0074	RSP_1304	CI/CI	O	922	71	0.643554	4.27036	0.60793	0.764089
**9**	RSP_0077	RSP_1324	CI/CI	O	231	71	0.719888	3.43494	-0.06281	0.410323
**10**	RSP_0079	RSP_1327	CI/CI	O	245	71	0.579103	3.44549	0.20524	0.594889
**11**	RSP_0082	RSP_1330	CI/CI	O	133	72	0.705639	2.98898	-0.07945	0.55382
**12**	RSP_0083	RSP_1331	CI/CI	O	128	69	0.707407	3.11253	0.21168	0.584358
**13**	RSP_0100	RSP_2512	CI/CI	O	125	64	0.618274	3.05805	-0.42671	0.807518
**14**	RSP_0102	RSP_2515	CI/CI	O	492	59	0.477429	3.76816	-0.62709	0.659012
**15**	RSP_0104	RSP_2518	CI/CI	O	424	62	0.525843	3.81648	-0.57334	0.739929
**16**	RSP_0105	RSP_2521	CI/CI	O	757	72	0.671845	4.27435	-0.66780	0.704801
**17**	RSP_0108	RSP_2525	CI/CI	O	184	69	0.557209	3.19609	-0.33265	0.769601
**18**	RSP_0112	RSP_2530	CI/CI	O	463	68	0.581917	4.08316	-0.37082	0.703236
**19**	RSP_0146	RSP_0889	CI/CI	O	112	38	1.007730	3.04369	0.88652	0.986234
**20**	RSP_0153	RSP_2225	CI/CI	O	224	57	0.436121	3.59200	0.18194	0.888719
**21**	RSP_0161	RSP_1883	CI/CI	I	365	31	0.187245	2.25486	0.37234	0.960136
**22**	RSP_0176	RSP_3074	CI/CII	O	603	72	0.588085	4.06177	0.40097	0.961756
**23**	RSP_0180	RSP_3166	CI/CII	O	282	70	0.644876	3.60665	0.28031	0.875611
**24**	RSP_0183	RSP_3462	CI/CII	O	493	64	0.569228	3.99432	0.23047	0.924721
**25**	RSP_0189	RSP_1149	CI/CI	O	460	69	0.609548	3.92983	0.53417	0.970659
**26**	RSP_0224	RSP_0658	CI/CI	O	761	69	0.832870	4.57555	0.47350	0.962005
**27**	RSP_0229	RSP_2238	CI/CI	O	520	69	0.587149	4.08485	0.54042	0.940408
**28**	RSP_0235	RSP_3050	CI/CII	I	341	35	0.227430	3.75074	-0.65361	0.369922

**29**	RSP_0248	RSP_0479	CI/CI	O	188	64	0.516304	3.14339	0.75035	0.962806
**30**	RSP_0254	RSP_1134	CI/CI	O	643	34	0.226694	4.25846	0.79379	0.950448
**31**	RSP_0287	RSP_2827	CI/CI	O	1136	73	0.730557	4.70011	0.40969	0.903622
**32**	RSP_0314	RSP_1556	CI/CI	I	51	6	0.025501	1.45070	-0.39029	0.628320
**33**	RSP_0329	RSP_3513	CI/CII	I	331	57	0.482733	3.78273	0.53095	0.976105
**34**	RSP_0345	RSP_3560	CI/CII	O	349	70	0.641227	3.62148	0.63831	0.962674
**35**	RSP_0382	RSP_1257	CI/CI	O	587	64	0.901936	3.82518	0.52019	0.961498
**36**	RSP_0413	RSP_4018	CI/PB	O	386	60	0.549848	3.80629	0.51687	0.965129
**37**	RSP_0423	RSP_3201	CI/CII	O	343	67	0.517721	3.72934	-0.19562	0.906776
**38**	RSP_0476	RSP_2364	CI/CI	I	216	29	0.180002	3.40030	-0.34370	0.382804
**39**	RSP_0480	RSP_0096	CI/CI	O	466	59	0.507366	4.06191	0.01518	0.810101
**40**	RSP_0563	RSP_2815	CI/CI	O	309	65	0.566510	3.71914	0.80608	0.986519
**41**	RSP_0576	RSP_3142	CI/CII	O	591	70	0.616942	4.01171	-0.53415	0.855879
**42**	RSP_0578	RSP_1736	CI/CI	O	217	54	0.387049	3.41171	0.21906	0.902215
**43**	RSP_0601	RSP_2410	CI/CI	O	295	53	0.471178	3.56268	0.41815	0.826388
**44**	RSP_0632	RSP_2155	CI/CI	O	438	62	0.541112	4.01125	0.68935	0.942882
**45**	RSP_0656	RSP_3330	CI/CII	I	592	36	0.234671	2.99804	0.73127	0.968786
**46**	RSP_0672	RSP_0920	CI/CI	O	291	60	0.571208	3.48936	0.70388	0.926450
**47**	RSP_0688	RSP_0550	CI/CI	O	428	68	0.645670	3.69382	0.58693	0.965885
**48**	RSP_0692	RSP_3027	CI/CII	I	473	33	0.219237	3.96259	-0.61582	0.869111
**49**	RSP_0698	RSP_2572	CI/CI	O	256	65	0.669324	3.67072	N/A	N/A
**50**	RSP_0723	RSP_0240	CI/CI	O	448	69	0.593561	3.91074	0.25612	0.809666
**51**	RSP_0759	RSP_4086	CI/PC	O	369	71	0.727604	3.85339	0.28560	0.875019
**52**	RSP_0766	RSP_0013	CI/CI	O	315	51	0.454135	3.61906	0.42140	0.927419
**53**	RSP_0772	RSP_1346	CI/CI	O	737	57	0.435067	4.09885	0.93844	0.971099
**54**	RSP_0817	RSP_2004	CI/CI	O	447	61	0.598539	3.91600	0.57781	0.920292
**55**	RSP_0840	RSP_1989	CI/CI	O	917	75	0.686148	4.28106	0.91017	0.989070
**56**	RSP_0893	RSP_1120	CI/CI	O	433	72	0.759936	4.04181	0.04014	0.924927
**57**	RSP_0902	RSP_0089	CI/CI	O	221	61	0.583574	3.61005	0.66210	0.913775
**58**	RSP_0945	RSP_3035	CI/CII	O	483	70	0.692597	4.07412	0.37295	0.920356
**59**	RSP_0953	RSP_2415	CI/CI	O	629	70	0.637515	4.01212	0.11350	0.951903

**60**	RSP_0961	RSP_2192	CI/CI	O	681	62	0.534460	4.15548	-0.38291	0.891503
**61**	RSP_0970	RSP_1771	CI/CI	O	302	65	0.552980	3.74750	0.35957	0.898710
**62**	RSP_0971	RSP_2392	CI/CI	I	185	48	0.326579	3.39940	0.44409	0.942510
**63**	RSP_0976	RSP_3147	CI/CII	O	594	68	0.617250	4.10615	-0.05216	0.954787
**64**	RSP_0979	RSP_3150	CI/CII	O	251	65	0.543268	3.18995	0.35558	0.908908
**65**	RSP_0990	RSP_3687	CI/CII	O	325	72	0.652188	3.62353	0.46991	0.966980
**66**	RSP_0992	RSP_3715	CI/CII	O	957	55	0.422896	4.58420	0.87909	0.974658
**67**	RSP_0993	RSP_3714	CI/CII	O	113	50	0.314391	2.94354	0.87215	0.964517
**68**	RSP_0994	RSP_3713	CI/CII	O	522	59	0.418185	4.15564	0.90304	0.981186
**69**	RSP_1013	RSP_2814	CI/CI	O	433	72	0.625699	3.94540	0.57443	0.962445
**70**	RSP_1016	RSP_1572	CI/CI	O	157	50	0.392085	3.17191	0.84385	0.941937
**71**	RSP_1036	RSP_1035	CI/CI	O	184	70	0.813752	1.82512	0.93167	0.991348
**72**	RSP_1097	RSP_1832	CI/CI	O	434	70	0.688764	3.87651	0.66925	0.958726
**73**	RSP_1109	RSP_2147	CI/CI	O	351	64	0.534060	3.67478	0.71439	0.963471
**74**	RSP_1123	RSP_3553	CI/CII	O	1052	70	0.645533	4.55461	0.66865	0.964935
**75**	RSP_1184	RSP_2660	CI/CI	O	326	67	0.643214	3.76786	0.54019	0.969391
**76**	RSP_1255	RSP_2305	CI/CI	O	310	49	0.419082	3.11167	-0.10193	0.76224
**77**	RSP_1260	RSP_6198	CI/CII	I	151	47	0.322542	3.19690	N/A	N/A
**78**	RSP_1261	RSP_4060	CI/PC	I	137	39	0.284662	3.21611	0.53485	0.946446
**79**	RSP_1272	RSP_2681	CI/CI	I	181	52	0.456059	3.30384	0.19479	0.934691
**80**	RSP_1278	RSP_2504	CI/CI	O	224	61	0.52650	3.57211	-0.17321	0.81441
**81**	RSP_1282	RSP_3271	CI/CII	O	473	69	0.647552	3.84736	0.91910	0.963447
**82**	RSP_1283	RSP_3270	CI/CII	O	357	21	0.126920	3.70433	0.83414	0.924013
**83**	RSP_1284	RSP_3267	CI/CII	I	291	13	0.068597	3.46547	0.82597	0.923523
**84**	RSP_1285	RSP_3266	CI/CII	I	332	32	0.233917	3.70860	0.91496	0.966171
**85**	RSP_1303	RSP_0080	CI/CI	O	444	75	0.657570	3.94795	0.31454	0.624023
**86**	RSP_1307	RSP_0076	CI/CI	O	367	57	0.499651	4.07544	-0.63186	0.327754
**87**	RSP_1309	RSP_0063	CI/CI	O	270	60	0.484854	3.56010	0.23342	0.555027
**88**	RSP_1312	RSP_0053	CI/CI	O	540	70	0.708251	4.18678	0.18128	0.601536
**89**	RSP_1319	RSP_0072	CI/CI	O	246	55	0.524494	3.67474	-0.22564	0.545402
**90**	RSP_1320	RSP_0034	CI/CI	O	696	64	0.525934	4.43753	-0.25300	0.478342

**91**	RSP_1322	RSP_0066	CI/CI	O	370	67	0.596330	3.95845	0.06703	0.579709
**92**	RSP_1326	RSP_0078	CI/CI	O	262	58	0.467581	3.54658	-0.17711	0.399428
**93**	RSP_1379	RSP_2899	CI/CI	O	484	62	0.546570	4.09397	0.68168	0.982727
**94**	RSP_1463	RSP_3181	CI/CII	O	387	70	0.611751	3.93457	0.64618	0.961892
**95**	RSP_1492	RSP_1869	CI/CI	O	404	65	0.557369	3.92617	0.90769	0.98851
**96**	RSP_1499	RSP_3272	CI/CII	O	560	71	0.652656	4.17906	0.21260	0.951588
**97**	RSP_1513	RSP_3585	CI/CII	O	405	55	0.447979	2.21730	0.30086	0.939961
**98**	RSP_1532	RSP_0196	CI/CI	O	427	54	0.613782	3.66485	-0.00349	0.913289
**99**	RSP_1551	RSP_0608	CI/CI	O	127	58	0.493332	3.30214	0.67871	0.919969
**100**	RSP_1574	RSP_0820	CI/CI	O	164	65	0.548452	3.29324	0.77904	0.922532
**101**	RSP_1591	RSP_3122	CI/CII	O	329	47	0.390695	3.71051	0.30788	0.943241
**102**	RSP_1613	RSP_1413	CI/CII	O	321	68	0.648508	3.77431	0.57819	0.990283
**103**	RSP_1614	RSP_1412	CI/CII	O	791	67	0.540984	4.33367	0.11322	0.95553
**104**	RSP_1638	RSP_2062	CI/CI	I	126	5	0.040646	0.46153	0.73685	0.760409
**105**	RSP_1645	RSP_3652	CI/CII	I	419	4	0.056517	0.58526	0.99327	0.969357
**106**	RSP_1647	RSP_3650	CI/CII	I	312	13	0.020664	0.78935	0.75067	0.980507
**107**	RSP_1650	RSP_2352	CI/CI	O	531	68	0.645887	3.95249	0.18626	0.817500
**108**	RSP_1653	RSP_6190	CI/CII	I	208	18	0.082103	1.09486	N/A	N/A
**109**	RSP_1662	RSP_2080	CI/CI	O	345	61	0.563715	3.89349	0.61863	0.907341
**110**	RSP_1696	RSP_2630	CI/CI	O	837	61	0.483571	4.21750	0.84520	0.986697
**111**	RSP_1706	RSP_1121	CI/CI	O	241	60	0.549276	3.52337	-0.27708	0.815809
**112**	RSP_1766	RSP_1848	CI/CI	O	495	65	0.566813	3.79728	0.55145	0.966212
**113**	RSP_1767	RSP_2323	CI/CI	O	250	63	0.610545	3.69283	0.35464	0.896008
**114**	RSP_1787	RSP_2334	CI/CI	O	321	63	0.588838	3.86455	0.44511	0.949694
**115**	RSP_1820	RSP_0088	CI/CI	O	168	47	0.396037	3.13828	0.75561	0.97212
**116**	RSP_1843	RSP_1053	CI/CI	O	449	67	0.547081	3.57513	0.83213	0.979609
**117**	RSP_1850	RSP_1786	CI/CI	O	793	65	0.702807	4.42668	0.83239	0.983226
**118**	RSP_1889	RSP_3093	CI/CII	O	242	51	0.402002	3.32604	0.30396	0.915223
**119**	RSP_1894	RSP_2496	CI/CI	I	148	19	0.091656	2.97837	0.83145	0.956095
**120**	RSP_1927	RSP_3172	CI/CII	O	275	61	0.511790	3.64482	0.43271	0.928566
**121**	RSP_1931	RSP_2653	CI/CI	O	292	69	0.636251	3.62235	0.42616	0.904744

**122**	RSP_1944	RSP_2820	CI/CI	O	248	58	0.542950	3.64774	0.10474	0.764086
**123**	RSP_1951	RSP_3622	CI/CII	I	116	4	0.024170	0.14281	0.21860	0.730684
**124**	RSP_1954	RSP_6191	CI/CII	I	84	40	0.317081	2.49821	N/A	N/A
**125**	RSP_1955	RSP_3647	CI/CII	I	219	28	0.207697	0.20902	0.76438	0.877883
**126**	RSP_1956	RSP_6196	CI/CII	I	124	8	0.039573	0.27507	N/A	N/A
**127**	RSP_1966	RSP_3007	CI/CII	I	242	1	0.027940	0.05770	0.96884	0.996295
**128**	RSP_1984	RSP_2284	CI/CI	O	371	70	0.746569	3.88954	0.82256	0.992951
**129**	RSP_1998	RSP_3049	CI/CII	O	402	62	0.578331	4.12153	-0.61255	0.230968
**130**	RSP_2061	RSP_1637	CI/CI	I	70	15	0.054750	0.22191	0.12300	0.580932
**131**	RSP_2063	RSP_1639	CI/CI	I	147	9	0.031705	0.30165	0.99949	0.970009
**132**	RSP_2064	RSP_6012	CI/CI	I	126	11	0.048522	0.92465	N/A	N/A
**133**	RSP_2065	RSP_1640	CI/CI	O	171	61	0.421863	3.27128	0.68874	0.964720
**134**	RSP_2098	RSP_2326	CI/CI	O	623	73	0.673511	4.10123	0.84743	0.975548
**135**	RSP_2106	RSP_2325	CI/CI	O	384	69	0.596186	3.79560	0.92326	0.992759
**136**	RSP_2122	RSP_3346	CI/CII	O	570	67	0.510323	3.30312	0.34124	0.817039
**137**	RSP_2124	RSP_3347	CI/CII	O	347	68	0.577281	3.73349	0.56388	0.926372
**138**	RSP_2184	RSP_3292	CI/CII	O	541	61	0.549624	4.10063	0.47979	0.955524
**139**	RSP_2189	RSP_2508	CI/CI	O	534	68	0.607157	3.95074	0.21921	0.768630
**140**	RSP_2201	RSP_2200	CI/CI	O	129	50	0.408903	3.01908	0.51505	0.947560
**141**	RSP_2227	RSP_2886	CI/CI	O	416	62	0.539259	3.84200	-0.21690	0.821566
**142**	RSP_2232	RSP_1001	CI/CI	O	367	61	0.541133	3.74396	0.73552	0.978663
**143**	RSP_2247	RSP_1708	CI/CI	O	687	72	0.648110	4.21820	0.64661	0.942124
**144**	RSP_2297	RSP_3935	CI/PA	O	505	54	0.400796	4.06519	-0.55511	0.771039
**145**	RSP_2360	RSP_2997	CI/CI	O	403	71	0.833229	4.21319	0.09133	0.772427
**146**	RSP_2397	RSP_3040	CI/CII	O	362	68	0.662673	3.71414	-0.04336	0.906989
**147**	RSP_2441	RSP_1589	CI/CI	I	588	74	0.787488	4.44180	-0.14437	0.594697
**148**	RSP_2459	RSP_2809	CI/CI	O	313	66	0.548626	3.66474	0.92413	0.987299
**149**	RSP_2470	RSP_2996	CI/CI	O	181	49	0.673937	3.66838	0.14258	0.858435
**150**	RSP_2482	RSP_4189	PC/PD	I	294	6	0.026956	0.56079	0.91622	0.925024
**151**	RSP_2501	RSP_3873	CI/PA	O	278	54	0.466219	3.88168	0.80268	0.968882
**152**	RSP_2513	RSP_0101	CI/CI	O	190	47	0.391735	3.09759	-0.56462	0.756161

**153**	RSP_2522	RSP_0106	CI/CI	O	332	57	0.472156	3.77278	-0.57882	0.792289
**154**	RSP_2523	RSP_0107	CI/CI	O	166	57	0.533028	2.95182	-0.45876	0.796510
**155**	RSP_2527	RSP_0110	CI/CI	O	386	66	0.537245	4.21251	-0.18680	0.756615
**156**	RSP_2565	RSP_1159	CI/CI	O	305	34	0.219552	3.66802	0.71559	0.982516
**157**	RSP_2592	RSP_2881	CI/CI	O	388	57	0.471463	3.98845	0.25824	0.948761
**158**	RSP_2607	RSP_3017	CI/CII	O	169	58	0.465102	3.19704	-0.20389	0.818699
**159**	RSP_2618	RSP_3826	CI/CII	O	256	65	0.612057	3.66259	0.87986	0.981221
**160**	RSP_2623N	RSP_2850	CI/CI	O	930	65	0.638109	4.36294	N/A	N/A
**161**	RSP_2673	RSP_2578	CI/CI	O	691	75	0.655391	4.03489	0.11597	0.941579
**162**	RSP_2749	RSP_0217	CI/CI	O	426	73	0.733817	4.00057	0.54474	0.945413
**163**	RSP_2779	RSP_2380	CI/CI	O	599	58	0.477310	3.89028	-0.38374	0.850766
**164**	RSP_2781	RSP_0899	CI/CI	O	172	64	0.584313	3.17757	0.45196	0.895072
**165**	RSP_2846	RSP_0154	CI/CI	O	293	64	0.551715	3.68567	0.70291	0.967964
**166**	RSP_2856	RSP_0704	CI/CI	O	624	61	0.517289	4.20314	0.86811	0.989641
**167**	RSP_2888	RSP_3341	CI/CII	O	145	62	0.477006	2.97673	0.95796	0.983533
**168**	RSP_2907	RSP_2977	CI/CI	O	363	58	0.506520	3.69699	0.88859	0.988763
**169**	RSP_2909	RSP_3743	CI/CII	O	321	60	0.497929	3.71394	0.63509	0.977449
**170**	RSP_3015	RSP_3969	CII/PB	O	308	44	0.329804	3.70467	0.46466	0.958512
**171**	RSP_3028	RSP_2984	CII/CI	O	407	46	0.359677	3.86678	0.57981	0.932895
**172**	RSP_3046	RSP_4118	CII/PC	O	310	52	0.447389	3.33577	-0.20693	0.233725
**173**	RSP_3098	RSP_0118	CII/CI	O	293	67	0.595911	3.60574	0.36872	0.909865
**174**	RSP_3106	RSP_4276	CII/PE	O	300	68	0.595143	3.72807	0.81053	0.851880
**175**	RSP_3113	RSP_2316	CII/CI	O	425	72	0.683283	4.07099	0.17551	0.765243
**176**	RSP_3116	RSP_3345	CII/CII	O	410	69	0.675768	3.86288	0.83015	0.991115
**177**	RSP_3134	RSP_3475	CII/CII	O	548	66	0.566951	4.02109	-0.59925	0.870252
**178**	RSP_3144	RSP_4010	CII/PB	O	336	48	0.360122	3.92080	-0.26808	0.912487
**179**	RSP_3187	RSP_0125	CII/CI	O	536	57	0.447032	4.08333	0.70809	0.976573
**180**	RSP_3253	RSP_3443	CII/CII	O	320	62	0.575325	3.71720	-0.28803	0.575458
**181**	RSP_3268	RSP_2956	CII/CI	O	665	42	0.304346	4.28667	0.25449	0.789881
**182**	RSP_3275	RSP_1024	CII/CI	O	331	55	0.469839	3.74790	0.30183	0.960488
**183**	RSP_3288	RSP_1444	CII/CI	O	285	69	0.604742	3.55400	0.35996	0.94116

**184**	RSP_3325	RSP_3719	CII/CII	I	503	42	0.289510	2.52994	-0.10224	0.868195
**185**	RSP_3342	RSP_1546	CII/CI	I	163	16	0.099181	2.97302	0.37530	0.948898
**186**	RSP_3349	RSP_4072	CII/PC	I	294	36	0.235649	3.53509	0.08612	0.880886
**187**	RSP_3350	RSP_4071	CII/PC	I	340	36	0.218020	3.75669	-0.02951	0.887346
**188**	RSP_3372	RSP_0097	CII/CI	O	372	68	0.669786	3.81051	0.17696	0.890970
**189**	RSP_3397	RSP_3515	CII/CII	O	346	71	0.652047	3.72637	0.49766	0.920566
**190**	RSP_3398	RSP_3657	CII/CII	O	218	63	0.564273	3.52437	0.81774	0.985118
**191**	RSP_3406	RSP_2904	CII/CI	O	325	61	0.516338	3.84079	0.81423	0.966895
**192**	RSP_3410	RSP_3386	CII/CII	O	318	70	0.692506	3.69268	0.48639	0.952299
**193**	RSP_3436	RSP_6165	CII/CII	O	296	63	0.576081	3.65107	N/A	N/A
**194**	RSP_3497	RSP_4069	CII/PC	O	727	69	0.656009	4.24264	0.72107	0.948256
**195**	RSP_3505	RSP_0090	CII/CI	O	315	69	0.696879	3.76661	-0.17505	0.899837
**196**	RSP_3547	RSP_2495	CII/CI	O	987	69	0.615415	4.23662	0.91117	0.977266
**197**	RSP_3574	RSP_2935	CII/CI	O	515	64	0.577381	4.32380	0.35191	0.933123
**198**	RSP_3608	RSP_4107	CII/PC	O	552	68	0.662650	4.21283	0.33309	0.920516
**199**	RSP_3624	RSP_3792	CII/CII	I	238	8	0.031810	0.84340	0.77059	0.945248
**200**	RSP_3627	RSP_3784	CII/CII	I	448	5	0.018009	0.16972	N/A	N/A
**201**	RSP_3628	RSP_3786	CII/CII	I	106	16	0.139983	0.35132	0.11965	0.848261
**202**	RSP_3664	RSP_3405	CII/CII	O	244	66	0.664541	3.48733	0.82760	0.976566
**203**	RSP_3695	RSP_0237	CII/CI	O	376	69	0.575432	3.90670	0.42343	0.948438
**204**	RSP_3697	RSP_3861	CII/PA	O	332	53	0.402597	3.67781	0.44463	0.829274
**205**	RSP_3703	RSP_1179	CII/CI	O	424	69	0.577648	3.78361	0.64119	0.945314
**206**	RSP_3843	RSP_0024	PA/CI	O	182	61	0.550560	3.35197	0.32586	0.926370
**207**	RSP_3884	RSP_3887	PA/PA	O	392	69	0.714393	4.05386	0.65575	0.927851
**208**	RSP_3894	RSP_3978	PA/PB	I	276	4	0.061994	0.10045	0.03151	0.939597
**209**	RSP_3904	RSP_7352	PA/PE	I	638	2	0.347877	1.04557	N/A	N/A
**210**	RSP_3906	RSP_2981	PA/CI	I	260	65	0.626281	3.65019	0.09268	0.693436
**211**	RSP_3908	RSP_4251	PA/PD	I	185	1	0.002260	0.02787	N/A	N/A
**212**	RSP_3955	RSP_0726	PB/CI	O	485	64	0.586129	4.06753	-0.00354	0.959866
**213**	RSP_3956	RSP_3940	PB/PB	I	325	65	0.509857	3.76807	0.60279	0.970402

**214**	RSP_3985	RSP_3920	PB/PA	I	470	54	0.391150	4.07772	-0.69853	0.719158
**215**	RSP_4008	RSP_2813	PB/CI	O	515	58	0.447920	4.03893	0.71513	0.978911
**216**	RSP_4021	RSP_7387	PB/PD	O	418	61	0.575299	4.26041	N/A	N/A
**217**	RSP_4022	RSP_4185	PB/PD	O	322	65	0.641723	3.90798	0.92184	0.983829
**218**	RSP_4050	RSP_4049	CI/CI	O	453	34	0.656834	3.96129	0.90928	0.9909
**219**	RSP_4053	RSP_0834	PC/CI	O	480	33	0.237894	4.13898	-0.57692	0.875944
**220**	RSP_4103	RSP_1428	PC/CI	I	473	59	0.527437	4.04967	0.28614	0.936994
**221**	RSP_4138	RSP_3902	PD/PA	I	265	4	0.015558	0.10280	N/A	N/A
**222**	RSP_4139	RSP_3901	PD/PA	I	270	39	0.289433	3.76069	0.84836	0.987637
**223**	RSP_4165	RSP_4182	PD/PD	O	428	57	0.406397	3.86125	0.32514	0.908499
**224**	RSP_4178	RSP_3012	PD/CII	I	119	26	0.162918	0.78856	0.57972	0.962900
**225**	RSP_4207	RSP_3721	PD/CII	I	379	34	0.224303	3.96258	-0.15449	0.624927
**226**	RSP_4209	RSP_3723	PD/CII	I	388	29	0.193555	2.23114	0.85116	0.989630
**227**	RSP_4252	RSP_3907	PD/PA	I	163	2	0.004987	0.07574	N/A	N/A
**228**	RSP_6015	RSP_6036	CI/CI	I	78	35	0.244177	2.93256	N/A	N/A
**229**	RSP_6035	RSP_3772	CI/CII	I	113	36	0.265381	0.94993	N/A	N/A
**230**	RSP_6194	RSP_6200	CII/CII	I	140	4	0.017398	0.47209	N/A	N/A
**231**	RSP_6234	RSP_6250	CII/CI	O	324	61	0.691169	3.34233	N/A	N/A
**232**	RSP_6256	RSP_6158	CI/CI	I	159	42	0.220655	2.75572	N/A	N/A
**233**	RSP_7246	RSP_4028	PC/PB	I	354	33	0.216009	1.50637	N/A	N/A
**234**	RSP_7390	RSP_3896	PD/PA	I	413	15	0.075829	0.69508	N/A	N/A

[a]gene name as shown in *R. sphaeroides* 2.4.1 annotation at NCBI
[b]location: chromosome I (CI), chromosome II (CII), or plasmids (PA,PB, PC, PD, or PE)
[c]paralog type: out-paralog (O) or in-paralog (I)
[d]average length of the duplicate genes
[e]percentage of amino acid divergence between protein homologs
[f]evolutionary constraint parameters: nonsynonymous substitution rate (K_a) and synonymous substitution rate (K_s)
[g]Pearson's correlation
[h]cosine similarity.
Note that "N/A" is given for the pair, whose corresponding gene expression does not exist in the microarray.
