# Supplementary material for: Evolutionary constraints and expression analysis of gene duplications in Rhodobacter sphaeroides 2.4.1
Source: BMC Res Notes. 2012 Apr 25;5:192. doi: 10.1186/1756-0500-5-192 (PMC3494609; doi:10.1186/1756-0500-5-192)

FIG. A1. Normalized gene expression panel of duplicated gene pairs in *R. sphaeroides* 2.4.1. Three replications of each growth condition are shown, with the following conditions: (1) 3W, (2) 10W, (3) 100W, (4) 10W DMSO, (5) Aerobic, (6) 2% Oxygen, (7) Dark DMSO. Green represents low levels of expression while red represents high levels of expression.

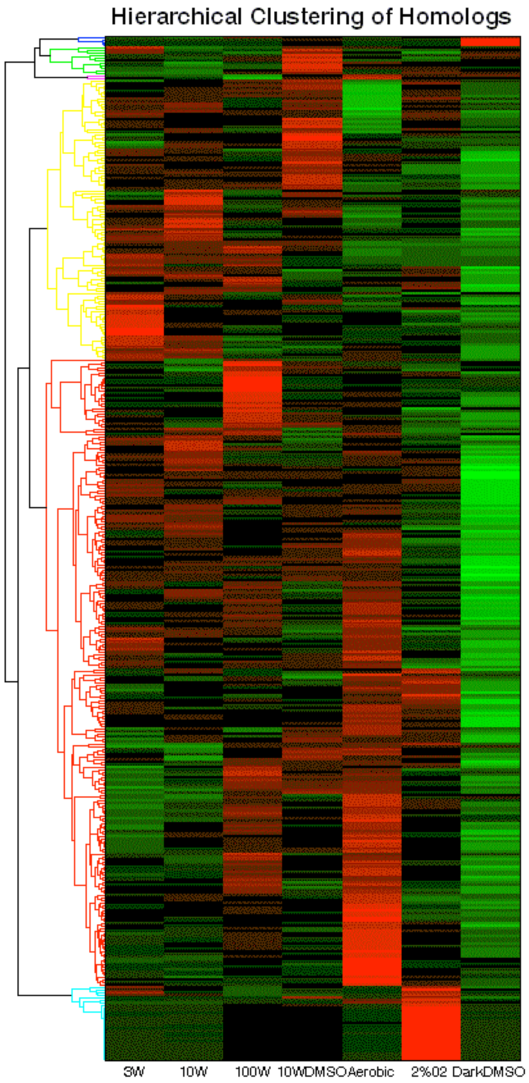

Supplement: Additional file 2 — Figure A1. Relationship between normalized correlation values and structural constraints on duplicated genes in R. sphaeroides. In-paralogs are shown in blue squares and out-paralogs are shown in red circles: (A) Ka of in-paralogs, (B) Ks of in-paralogs, (C) Ka of out-paralogs, and (D) Ks of out-paralogs. [file 1756-0500-5-192-S2.pdf]
