# Supplementary material for: Evolutionary constraints and expression analysis of gene duplications in Rhodobacter sphaeroides 2.4.1
Source: BMC Res Notes. 2012 Apr 25;5:192. doi: 10.1186/1756-0500-5-192 (PMC3494609; doi:10.1186/1756-0500-5-192)

FIG. A2. Relationship between normalized correlation values and structural constraints on duplicated genes in *R. sphaeroides*. Out-paralogs are shown in red circles and in-paralogs are shown in blue squares: (A) In-paralog K_a, (B) In-paralog K_s, (C) In-paralog ω, (D) Out-paralog K_a, (E) Out-paralog K_s, and (F) Out-paralog ω.

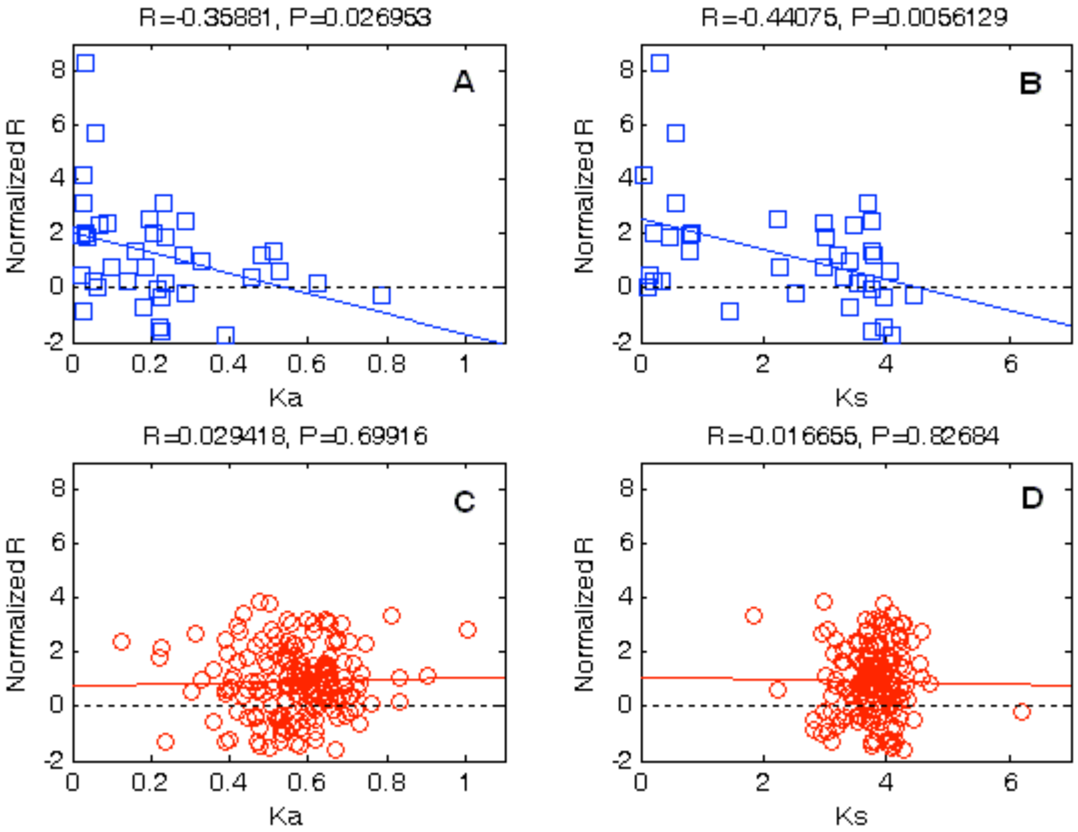

Supplement: Additional file 3 — Figure A2. Hierarchical clustering of the normalized expressions of homologs in R. sphaeroides 2.4.1. Each column represents the following growth condition: (1) 3W, (2) 10W, (3) 100W, (4) 10W DMSO, (5) Aerobic, (6) 2% Oxygen, and (7) Dark DMSO. Three replications of each growth condition are averaged and the averaged expression levels are normalized by z-score transformation before clustering with hierarchical clustering. Green represents low levels of expression while red represents high levels of expression. [file 1756-0500-5-192-S3.pdf]
